# PPARG: A Novel Target for Yellow Tea in Kidney Stone Prevention

**DOI:** 10.3390/ijms241511955

**Published:** 2023-07-26

**Authors:** Mingjie Su, Siyao Sang, Taotao Liang, Hui Li

**Affiliations:** 1 Human Phenome Institute, Zhangjiang Fudan International Innovation Center, Fudan University, Shanghai 200438, China; 2 MOE Key Laboratory of Contemporary Anthropology, Fudan University, Shanghai 200438, China; 3 Department of Hematology, Affiliated Tumor Hospital of Zhengzhou University, Henan Cancer Hospital, Zhengzhou 450008, China; 4 Fudan-Datong Institute of Chinese Origin, Shanxi Academy of Advanced Research and Innovation, Datong 037006, China

**Keywords:** yellow tea, kidney stones, PPARG, oxidative stress, fibrosis

## Abstract

Kidney stones are a common urological disorder with increasing prevalence worldwide. The treatment of kidney stones mainly relies on surgical procedures or extracorporeal shock wave lithotripsy, which can effectively remove the stones but also result in some complications and recurrence. Therefore, finding a drug or natural compound that can prevent and treat kidney stones is an important research topic. In this study, we aimed to investigate the effects of yellow tea on kidney stone formation and its mechanisms of action. We induced kidney stones in rats by feeding them an ethylene glycol diet and found that yellow tea infusion reduced crystal deposits, inflammation, oxidative stress, and fibrosis in a dose-dependent manner. Through network pharmacology and quantitative structure–activity relationship modeling, we analyzed the interaction network between the compounds in yellow tea and kidney stone-related targets and verified it through in vitro and in vivo experiments. Our results showed that flavonoids in yellow tea could bind directly or indirectly to peroxisome proliferator-activated receptor gamma (PPARG) protein and affect kidney stone formation by regulating PPARG transcription factor activity. In conclusion, yellow tea may act as a potential PPARG agonist for the prevention and treatment of renal oxidative damage and fibrosis caused by kidney stones.

## 1. Introduction

Kidney stones are a common urological disorder with an increasing prevalence worldwide. The formation of kidney stones involves multiple factors, such as genetics, metabolism, diet, infection, medication, etc. [1,2]. Oxalate stones are the most prevalent type of kidney stones, accounting for over 80% of cases. The formation of oxalate stones is related to the deposition of oxalate and calcium ions in the urine, which is influenced by various factors, such as urine pH, urine volume, concentration of other solutes in the urine, etc. [3].

Currently, the treatment of kidney stones mainly relies on surgical procedures or extracorporeal shock wave lithotripsy, which can effectively remove the stones but also result in some complications and recurrence [4]. Therefore, finding a drug or natural compound that can prevent and treat kidney stones is an important research topic. Recently, more and more studies have shown that some natural compounds have anti-urolithic activity, such as tea polyphenols, flavonoids, citrate, etc. These compounds can prevent or dissolve kidney stones by affecting the deposition of oxalate and calcium ions in the urine, inhibiting oxidative stress and fibrosis, regulating apoptosis and epithelial–mesenchymal transition (EMT), etc. [5].

Yellow tea is a special type of tea that undergoes a “yellowing” process during its production, which inactivates the polyphenol oxidase in the tea leaves and preserves a high content of polyphenols and other active ingredients [6]. Yellow tea has various physiological effects, such as lowering blood pressure and blood lipids, antioxidation, antibacterial, etc. Our previous research results showed that compared with five other types of tea, the water extract of fully dehydrogenated yellow tea could effectively lower the blood glucose level of high-fat diet-induced type 2 diabetes mellitus (DM2) mice, reduce the number of lipid droplets in their liver, inhibit liver injury, and alleviate their liver and systemic inflammation levels. The results of Sang et al. showed that yellow tea significantly reduced uric acid levels in hyperuricemia rats and protected the liver and kidney by regulating inflammatory response, autophagy, and apoptosis [7].

Based on these findings, we hypothesized that yellow tea could prevent renal oxalate stones and that its mechanism might be related to its compounds interacting with kidney stone-related targets. To test this hypothesis, we used methods such as synergistic network [8] pharmacology [9] and quantitative structure–activity relationship (QSAR) modeling [10] to analyze the interaction network between the compounds in yellow tea and kidney stone-related targets from a systems biology perspective and verified it by in vitro and in vivo experiments.

## 2. Results

### 2.1. The Effects of Yellow Tea on Kidney Stone Formation in Rats

#### 2.1.1. Yellow Tea Inhibits Renal Crystal Deposition and Oxidative Stress in Rats

**Figure 1:**
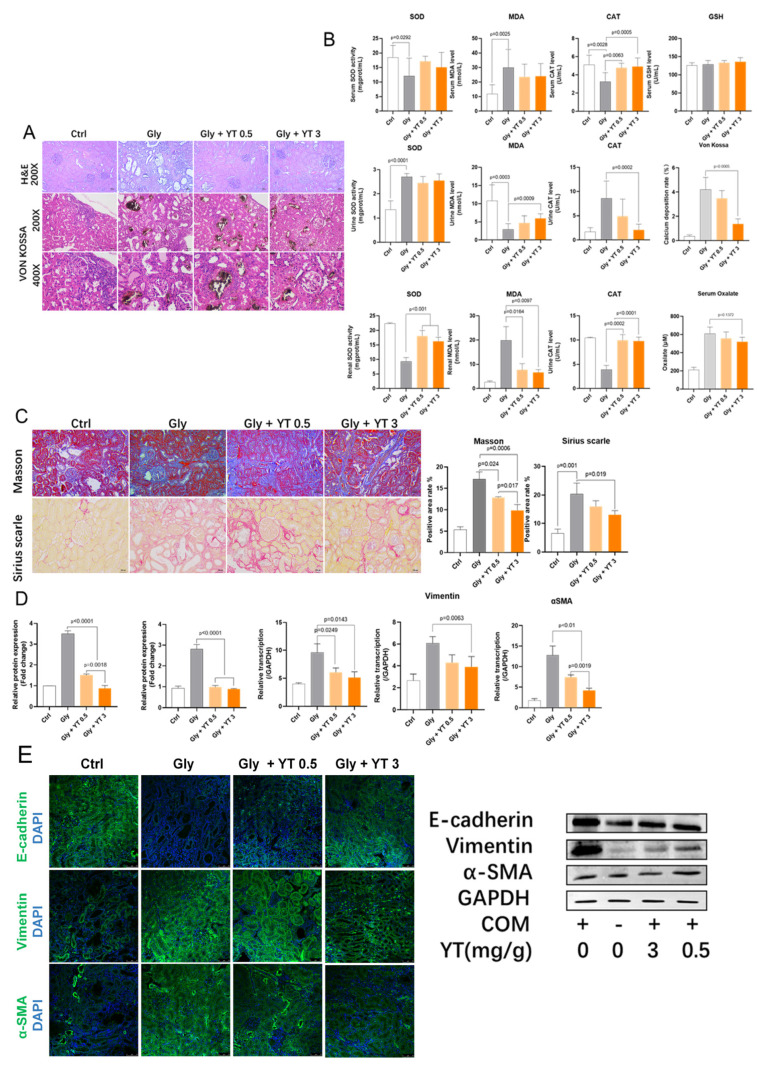
Yellow tea reduces oxidative damage, fibrosis, and epithelial–mesenchymal transition in rat kidneys with nephrolithiasis. (**A**). H&E and Von Kossa staining of rat kidney sections from control (Ctrl), ethylene glycol (Gly), and ethylene glycol plus low (0.5 g/dL) or high (3 g/dL) yellow tea concentration (Gly+YT0.5 and Gly+YT3) groups (n = 8). Black indicates calcium oxalate deposition. Dose-dependent reduction of deposition by yellow tea. (**B**). SOD, MDA, CAT, and GSH concentrations in plasma and urine. Bar graphs show the mean ± SEM. Suppression of oxidative stress by yellow tea. (**C**). Masson’s trichrome and Sirius red staining and positive area quantification. Inhibition of fibrosis by yellow tea. (**D**). RT-qPCR results for fibronectin, collagen I, E-cad, Vim, and α-SMA. Bar graphs show the mean ± SEM. Downregulation of fibrosis-related and EMT-related genes by yellow tea. (**E**). Immunofluorescence (left) and Western blot (right) results for E-cad, Vim, and α-SMA. Yellow tea decreases mesenchymal markers and increases epithelial markers.

Kidney stones were induced in rats by feeding them an ethylene glycol diet. We observed calcium oxalate crystal deposits in the dilated tubules of the kidney, as well as tubular epithelial cell damage and inflammatory cell infiltration. The levels of oxidative stress markers (MDA, SOD, and CAT) were measured in kidney, serum, and urine samples. We found that the yellow tea infusion reduced crystal deposition, inflammation, oxidative stress, and fibrosis in a dose-dependent manner (Figure 1A–C). The yellow tea infusion also lowered the plasma oxalate concentration, but the difference was not statistically significant (Figure 1B).

#### 2.1.2. Yellow Tea Alleviates Renal Fibrosis in Rats

Kidney stones can cause kidney injury and fibrosis. The expression of collagen fibers and fibrotic genes (fibronectin and collagen 1) in the kidney tissue were assessed. We found that ethylene glycol-induced kidney stone rats had increased collagen fiber deposition and fibrotic gene transcription compared to control rats. Yellow tea infusion significantly reversed these changes in a dose-dependent manner (Figure 1D).

#### 2.1.3. Yellow Tea Inhibits Renal Epithelial–Mesenchymal Transition in Rats

Epithelial–mesenchymal transition (EMT) is a process that contributes to kidney fibrosis under stress conditions such as calcium oxalate crystals. The expression of epithelial marker (E-cadherin) and mesenchymal markers (vimentin and α-SMA) in the kidney tissue were evaluated. We found that ethylene glycol-induced kidney stone rats had reduced E-cadherin expression and increased vimentin and α-SMA expression compared with control rats. Yellow tea infusion inhibited EMT activation in a dose-dependent manner (Figure 1E).

### 2.2. Yellow Tea Compound Target Screening Results

#### 2.2.1. Representative Molecules of Yellow Tea and Their Target Network

**Figure 2:**
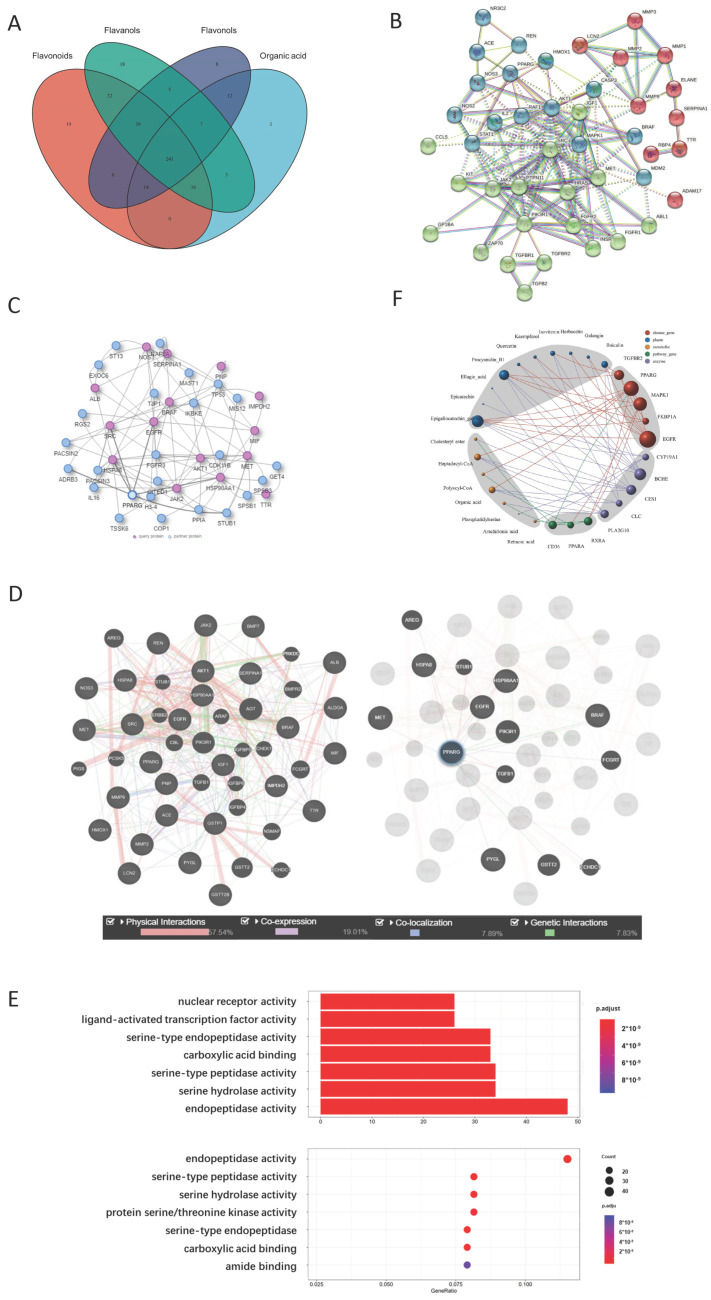
Yellow tea compounds target nephrolithiasis-related proteins based on network pharmacology methods. Target proteins of the four major classes of compounds in yellow tea infusion and nephrolithiasis-related proteins were analyzed by network pharmacology methods. (**A**). Venn diagram of overlapping proteins (241) between yellow tea compound targets and nephrolithiasis-related targets. (**B**). Interaction network of yellow tea compound target proteins and nephrolithiasis-related kidney disease proteins (score > 0.4 in STRING, medium confidence). PPARG and other genes are at the center of the network. (**C**). Interaction network of target proteins of 10 major compounds in yellow tea infusion with a score > 0.5 and key kidney disease proteins (in IID). Purple circles represent query proteins; blue circles represent interacting proteins. (**D**). Interaction network of target proteins of 10 major compounds in yellow tea infusion with a score > 0.5 and key kidney disease proteins (from GeneMANIA). The color of the line represents the specific type of protein interaction. IID and GeneMANIA results verified PPARG as an important protein in yellow tea acting on nephrolithiasis. (**E**). GO enrichment results of the intersection of target proteins of 10 compounds in yellow tea infusion and nephrolithiasis-related disease target proteins. The nuclear transcription factor pathway had a high score. (**F**). Interaction network of the intersection of yellow tea compound target proteins, all PPAR-related proteins, and all disease-related proteins with a binding score > 0.9. Yellow tea compounds, especially isovitexin, regulate TGF-β1 and other genes by directly acting on PPARG and CLC proteins.

Ten compounds from the yellow tea infusion were screened based on their molecular weight, drug-like score, and bioavailability score. These compounds included flavanols, flavonoids, flavonols, and organic acids (tannic acid). The structures of the above molecules were identified using PubChem. The protein targets of the above molecules were predicted based on their binding scores. We found that most of the high-scoring (>0.9) protein targets were associated with flavonoids (baicalin, isovitexin) and flavanols (EGCG) (Table 1). There were 241 protein targets that could interact with these four categories of compounds (Figure 2A).

#### 2.2.2. PPARG Is the Center of the Yellow Tea and Kidney Disease Target Network

The PPI network was constructed based on the protein targets of yellow tea compounds and the genes related to urolithiasis, chronic kidney disease, and renal fibrosis. We found that yellow tea compound targets did not include some important genes for these diseases, such as PKD1, PKD2, and UMOD. However, we noticed that VDR, a gene closely related to urolithiasis, was present in the network. Eight out of ten yellow tea compounds could bind to VDR, but with low binding scores (<0.5). We also observed that PPAR family proteins (PPARA and PPARG) were frequently involved in different clusters of the network, interacting with proteins such as NOS, SOD, CAT or ACE, ATK, SRC, and MAPK. Nine out of ten yellow tea compounds could bind to PPARG with high efficiency (>0.9). We validated the PPI network using the IID and GeneMANIA databases. We found consistent results with the STRING database. HSP90AA1, PPARG, and SRC were common proteins in both the IID and GeneMANIA networks (Figure 2B–D). 

#### 2.2.3. Metabolite Target Network Construction Based on PPARG Protein

The metabolites related to PPARG and its major interacting proteins (CD36, TGF-β1, and RXRA) were searched using the human metabolome database. We found that the PPARG gene has 197 experimentally validated interacting proteins and 42 functionally related intracellular or exogenous natural metabolites. Most of these metabolites are long-chain fatty acid metabolism intermediates or organic acids produced by cellular metabolism. These metabolites had 4533 target proteins in total. We focused on some key metabolites such as cholesterol ester, heptanoyl-CoA, polyacyl coenzyme A, organic acids, phosphatidylserine, arachidonic acid, and all-trans retinoic acid for the research (Table 2).

#### 2.2.4. GO Analysis Shows That Yellow Tea Affects Kidney Stones through Ligand-Activated Transcription Factor Activity

GO analysis was performed for all target proteins of the ten major compounds in yellow tea. We found 212 enriched biological functions (*p* < 0.05) including nuclear receptor activity, ligand-activated transcription factor activity, transmembrane receptor protein kinase activity, transmembrane receptor, protein tyrosine kinase activity, protein tyrosine kinase activity, etc. These functions suggested that yellow tea compounds may affect cell surface signal transduction, lipid metabolism, and nuclear receptor signaling pathways (Figure 2E).

#### 2.2.5. Network Analysis Shows That Isovitexin and Other Flavonoids Synergistically Act on PPARG Protein

The direct and indirect relationships between yellow tea compounds and PPAR family-related pathway proteins were performed using Cytoscape software (version 3.10.0, https://cytoscape.org/, accessed on 18 July 2023). We found that isovitexin and other flavonoids (baicalin, quercetin, kaempferol, galangin, naringenol) could bind to PPARG directly and indirectly through EGFR, AURKA, HSP90AA1, and FKBP1A. These proteins could also regulate the expression levels of metabolites related to PPARG such as cholesterol ester, heptanoyl-CoA, polyacyl coenzyme A, organic acids, phosphatidylserine, arachidonic acid, and all-trans retinoic acid. These metabolites could further affect PPAR pathway function through CD36, TGF-β1, RXRA, etc. These results suggested that yellow tea flavonoids may have synergistic effects on PPARG activation (Figure 2F).

### 2.3. QSAR Evaluation Shows That Catechins Have High PPARG Agonist Scores

The log EC50 (pEC50) of different PPARG agonists from ChEMBL were compared and analyzed, including the logP, the number of hydrogen bond acceptors (NumHAcceptors), the number of hydrogen bond donors (NumHDonors), and the molecular weight (MW) of active and inactive molecules based on Lipinski’s rule of five. We observed a high-density distribution of PPARG agonists in the MW range of about 400 to 500 Da and ALogP from −1 to 10. There was no significant difference in ALogP, NumHAcceptors, and NumHDonors between PPARG agonists and inhibitors, suggesting that the tertiary structure of the molecules might be the reason for the specific agonistic effect of agonists on PPARG protein. On the other hand, there was no significant difference in the distribution of LogP and MW between PPARG agonist molecules and non-agonist molecules.

A dataset containing 3295 compounds was used to build a random forest model. The dataset was split using the 80/20 data splitting rule into the learning and validation datasets, respectively, and a random forest model was trained with the learning dataset. The validation dataset experiment showed a linear distribution between observed and predicted pEC50. The predicted pEC50 of flavonoid small molecules based on this model are shown in Table 3. Isovitexin, a representative flavonoid compound, had a similar pEC50 for PPARG protein as a commercialized PPARG agonist (pioglitazone) (Table 3).

### 2.4. Flavonoids Regulate PPARG Transcription in HK-2 Cells

#### 2.4.1. Flavonoid Pretreatment Increased Cell Viability after H_2_O_2_ Stimulation

**Figure 3:**
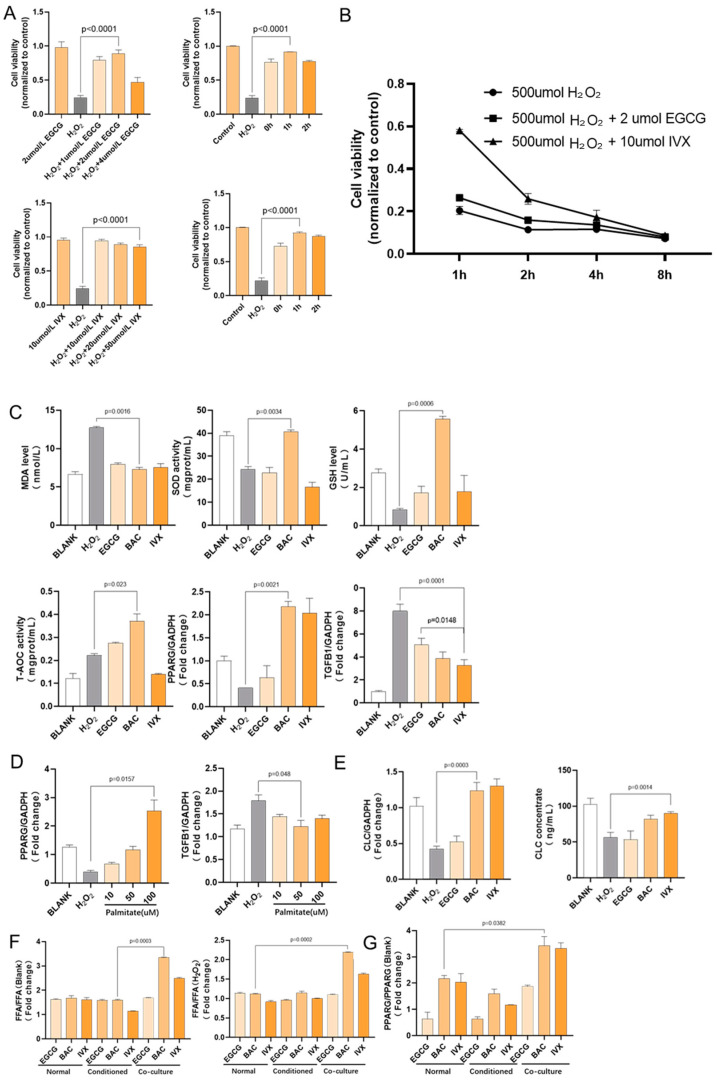
Effects of flavonoid compounds on oxidative markers and PPARG protein expression in HK-2 cells in vitro. HK-2 cells were treated with H_2_O_2_ and EGCG/IVX alone or in combination. (**A**). Cell viability at different concentrations of EGCG and IVX and incubation times. Optimal viability was observed with 2 µM EGCG and 10 µM IVX for 1 h. (**B**). Cell viability after drug treatment for different incubation times. Largest difference after 1 h of treatment. (**C**). MDA, GSH, SOD, T-AOC activities and PPARG and TGF-Β1 gene transcription after flavonoid pretreatment and H_2_O_2_ stimulation. Inhibition of oxidation by flavonoids. (**D**). PPARG and TGF-Β1 gene transcription after fatty acid (palmitate) treatment and H_2_O_2_ stimulation. Upregulation of PPARG and downregulation of TGF-Β1 by palmitate. (**E**). CLC gene transcription after flavonoid pretreatment and H_2_O_2_ stimulation of EOS cells. Left: RT-qPCR results; right: ELISA results. Upregulation of CLC by flavonoids. (**F**). Free fatty acid concentration under different culture conditions and flavonoid and H_2_O_2_ treatment. Higher free fatty acid level under co-culture conditions with flavonoids. (**G**). PPARG gene transcription under different culture conditions and flavonoid and H_2_O_2_ treatment. Higher PPARG transcription level under co-culture conditions with flavonoids. All bar graphs show the mean ± SEM, n = 6. IVX = isovitexin, BAC = Baicalin.

HK-2 cells treated with H_2_O_2_ were assigned to different groups according to different pretreatment concentrations of two flavonoids, EGCG and isovitexin. The results showed that treatment with EGCG or isovitexin alone had no significant effect on cell viability, while 2 µmol/L EGCG and 10 µmol/L isovitexin treatment for 1 h had the best protective effect against cell damage induced by H_2_O_2_; moreover, the maximum difference in cell viability between the H_2_O_2_+flavonoid group and H_2_O_2_ group was observed after 1 h of stimulation. In the following studies, we used 2 µmol/L EGCG and 10 µmol/L isovitexin pretreatment for 1 h followed by H_2_O_2_ stimulation for 1 h as the drug administration method. The concentrations of the other drugs that were used in this study are based on those used by published articles using HK-2 or epithelial cells (Figure 3A,B).

#### 2.4.2. Flavonoid Pretreatment Upregulated PPARG and Downregulated TGF-Β1 Gene Transcription

MDA and GSH levels, and SOD and T-AOC activity reflect the level of oxidative stress in cells. H_2_O_2_ treatment greatly induced oxidative stress. The experiments showed that after treatment with 500 µmol/L H_2_O_2_ for one hour, HK-2 cells pretreated with baicalin, EGCG, and isovitexin had lower MDA levels and significantly higher SOD activity, GSH levels, and T-AOC activity than HK-2 cells without flavonoid pretreatment. At the same time, HK-2 cells treated with these three flavonoids had a significantly increased PPARG gene transcription level and significantly decreased TGF-Β1 transcription level (Figure 3C).

#### 2.4.3. Fatty Acid Treatment Upregulated PPARG Gene Transcription in HK-2 Cells

After treatment with 500 µmol/L H_2_O_2_ for one hour, HK-2 cells pretreated with palmitate had higher PPARG transcription levels than HK-2 cells without flavonoid pretreatment. At the same time, the PPARG transcription level also increased in a dose-dependent manner. HK-2 cells pretreated with different concentrations of fatty acids had a significantly decreased TGF-Β1 transcription level (Figure 3D).

#### 2.4.4. Flavonoid Pretreatment Upregulated CLC Gene Transcription in Co-Cultured Eosinophils

CLC is considered to be a lysophospholipase homolog that has lysophospholipase activity and interacts with lysophospholipase. CLC protein’s lysophospholipase activity results in the formation of alternative cleavage products (GPC) from the substrate lysophosphatidylcholine. We detected CLC gene transcription in Eos cells in a co-culture system by RT-qPCR. The results showed that H_2_O_2_ stimulation significantly downregulated CLC gene transcription levels in Eos cells, while baicalin and isovitexin significantly upregulated CLC gene transcription and protein expression levels in Eos cells. EGCG did not demonstrate the ability to regulate CLC gene transcription (Figure 3E).

#### 2.4.5. Flavonoid Pretreatment Upregulated Free Fatty Acid Concentration in Co-Cultured HK-2 Cells

CLC expresses endogenous lysophospholipase activity, releasing free palmitate from the substrate lysophosphatidylcholine. We detected the free fatty acid concentrations in the medium from single culture Eos and Eos/HK-2 co-culture. The results showed that under the action of H_2_O_2_ and different flavonoid compounds, the free fatty acid concentration in HK-2 cells increased to different extents compared with the controls without intervention or only treated with H_2_O_2_. When treated with baicalin or isovitexin, the free fatty acid level in HK-2 cells co-cultured with Eos was significantly higher than that in the conventional culture and conditional culture drug groups. EGCG did not increase the free fatty acid level in HK-2 cells co-cultured with Eos (Figure 3F).

#### 2.4.6. Co-Cultured HK-2 Cells Had a Higher PPARG Transcription Level

RT-qPCR analysis showed that H_2_O_2_ stimulation significantly reduced PPARG gene transcription in HK-2 cells, which is consistent with PPARG gene transcription and expression changes observed in the kidney tissue of nephrolithiasis rats (details are discussed in Section 2.5). After treatment with baicalin and isovitexin, the PPARG gene transcription level in HK-2 cells co-cultured with Eos was significantly higher under H_2_O_2_ stimulation (Figure 3G).

### 2.5. Yellow Tea Regulates PPARG Transcription Factor Activity

#### 2.5.1. Yellow Tea Regulated Kidney PPARG Pathway Protein Transcription in Nephrolithiasis Rats

**Figure 4:**
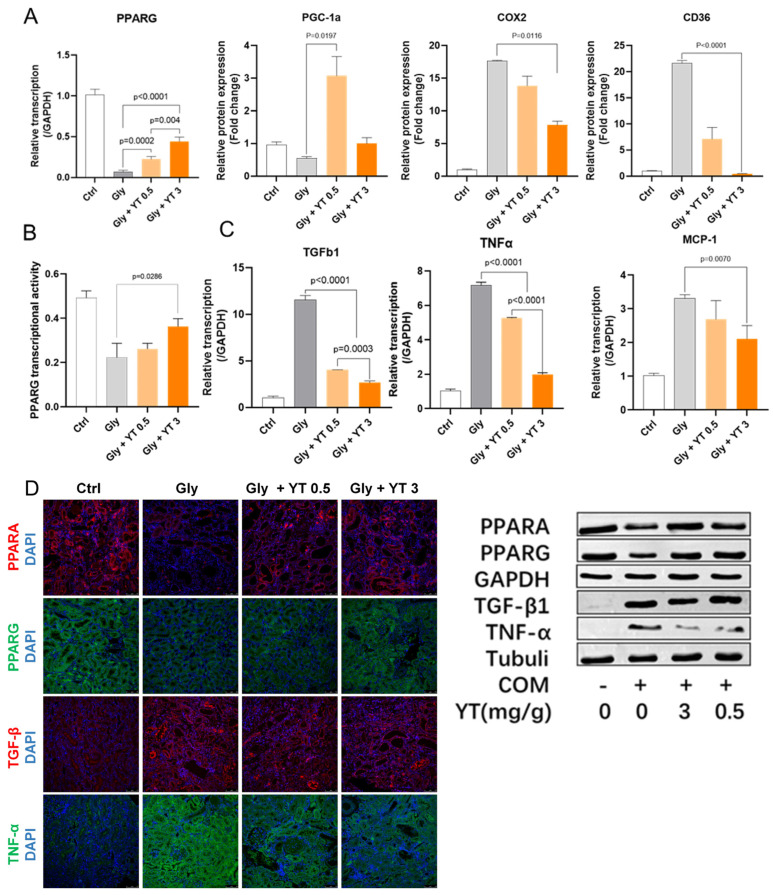
Yellow tea regulates PPARG function in nephrolithiasis rats. Rats with nephrolithiasis were gavaged with different concentrations of yellow tea. (**A**). PGC1a, COX2, and CD36 gene transcription in rat kidneys was regulated by yellow tea. (**B**). PPARG transcription factor activity in rat kidneys was upregulated by yellow tea. (**C**). TGF-Β1, TNFα, and MCP-1 transcription levels in rat kidneys inhibited by yellow tea. (**D**). PPARA, PPARG, TGF-Β1, and TNFα immunofluorescence (left) and Western blot results (right) showed upregulation of PPARG and downregulation of TGF-Β1 and TNFα by yellow tea. All bar graphs show the mean ± SEM, n = 8.

The transcription levels of PPAR family proteins, PPARG upstream proteins, kidney disease-related proteins, and EMT-related TGF-Β1 protein in rat kidney tissues from each experimental group were detected by RT-qPCR. The results showed that yellow tea gavage significantly upregulated PPARG gene transcription levels in nephrolithiasis rats and showed a significant dose accumulation effect. Yellow tea gavage also affected the transcription levels of PGC-1α, COX2, and CD36, which are related to PPARG function (Figure 4A).

#### 2.5.2. Yellow Tea Inhibited Downregulation of PPARG Transcription Factor Activity in Nephrolithiasis Rat Kidney

An assay based on PPRE sequence oligonucleotide probes was used to detect the PPARG transcription factor activity of extracted nuclear proteins. The results showed that nephrolithiasis caused a significant downregulation of PPARG protein nuclear transcription factor activity in rat kidney tissue cells, and yellow tea administration significantly inhibited this effect. There was a dose-dependent effect of yellow tea gavage on protection of PPARG protein transcription factor activity (Figure 4B).

#### 2.5.3. Yellow Tea Inhibited Inflammation- and Fibrosis-Related Cytokine Transcription in Nephrolithiasis Rat Kidney

PPAR’s function has been shown to be related to various inflammatory and autoimmune diseases. We detected TGF-Β1, TNFα, and MCP-1 transcription levels in rat kidney tissues from each experimental group. The results showed that TGF-Β1, TNFα, and MCP-1 transcription levels increased significantly in rat kidney tissue from the nephrolithiasis group, while yellow tea gavage significantly inhibited these levels in a dose-dependent manner (Figure 4C).

#### 2.5.4. Yellow Tea Affected Protein Expression in Nephrolithiasis Rats

PPARA, PPARG, TGF-β1, and TNF-α were selected among these processes, and their expression levels were detected by immunofluorescence staining and Western blot. The results showed that a high concentration of the yellow tea gavage could significantly activate the PPARA gene and PPARG gene expression and inhibit TGF-Β1 and TNF-α expression. The immunofluorescence staining, Western blot, and qRT-PCR results were consistent (Figure 4D).

## 3. Discussion

Nephrolithiasis is a urinary system disease that has plagued humanity for thousands of years [11,12]. Advanced medical technologies such as extracorporeal shock wave lithotripsy (ESWL) and ureteroscopy can only reduce the pain and health burden of stones that have already been formed rather than preventing the formation of stones [13]. These drawbacks make the recurrence rate of nephrolithiasis still high, and it increases by nearly 50% within 5–10 years after the initial stone formation [14]. Inhibiting the development of renal inflammatory fibrosis and improving the crystal clearance rate of the kidneys are key means to control nephrolithiasis in the long term. Previous studies have shown that oxidative stress and fibrosis are involved in the occurrence and recurrence of nephrolithiasis. At the same time, EMT, which is an important source of inflammation and fibrosis, has attracted the attention of researchers, but the regulatory mechanism of this process in the process of stone formation is still unclear [15]. Oxalate and calcium oxalate crystals can induce EMT and participate in the development of fibrotic tissue in nephrocalcinosis [16].

In several previous studies, flavonoids from different sources significantly improved renal injury in animal models of nephrolithiasis and chronic kidney disease [16,17]. In this study, we demonstrated that yellow tea gavage also improved the renal health status of ethylene glycol-induced nephrolithiasis rats. Under the same condition of ethylene glycol gavage, yellow tea gavage group rats had relatively lower plasma oxalate levels. The results of our study showed that yellow tea administration reversed the increase in renal MDA levels and increased the levels of SOD, GSH, and CAT, which is consistent with the results of previous studies on flavonoids.

The potential mechanism of yellow tea protection involves anti-oxidation, anti-fibrosis, and EMT inhibition [18]. Therefore, yellow tea may be able to act as a potential EMT inhibitor for the prevention and treatment of renal oxidative damage and fibrosis caused by nephrolithiasis, but its specific target is still unknown.

The specific compound composition of yellow tea soup was further detected and analyzed by non-targeted metabolomics based on LC-MS [19]. Based on network pharmacology combined with metabolomics, we studied the interaction between these compounds and targets. We discussed the specific targets of specific types of natural compounds in yellow tea that affect phenotypes related to nephrolithiasis.

We found that oxidative damage and fibrosis occurred in the kidney tissues of nephrolithiasis rats during the development of nephrolithiasis. EMT occurred in kidney tissue cells at the same time that PPAR family protein expression decreased significantly. We found that there are obvious shortcomings in defining kidney disease with limited clinical phenotypes. Therefore, we combined nephrolithiasis with chronic kidney disease and renal fibrosis, which have similar phenotypes in research models. We discussed and analyzed the influence of yellow tea compounds on the common genes between these three diseases.

Although yellow tea compounds cover a wide range of targets [19], they could not bind to all disease-related proteins and signaling pathways with high binding scores. In this case, we designed a two-layer target network combining proteins that interact with yellow tea target proteins and metabolites to expand our research scope on yellow tea regulation research objects. We successfully found an indirect regulation mode for PPARG based on metabolites that could not be found by simply studying compound–protein interactions. The representative flavonoids in yellow tea, baicalin, isovitexin, and EGCG, may directly affect the expression and transcriptional activity of PPARG protein, and also affect the expression and activity of CLC protein in eosinophils. For this finding to be clinically relevant, there must be eosinophil infiltration into nephrolithiasis tissue.

Results obtained by computational methods were verified based on more reliable methods. Specifically, we used the Pub-Chem fingerprint as an object for PPARG agonist activity and confirmed the compound’s molecular linear structure features. We used a random forest model to model and evaluate the flavonoid molecules’ PPARG protein agonist ability based on this model. We used molecular docking methods to verify baicalin’s ability to bind with CLC protein. We verified eosinophil infiltration in our rat stone model kidney by histological methods.

In this study, CLC lysophospholipase [18] was identified as an eosinophil-specific phospholipase that is involved in the decomposition of eosinophil-associated phosphatidylcholine into fatty acids, indirectly regulating the concentration of fatty acids in co-cultured HK-2 cells, thereby affecting the PPARG pathway process. Compared with flavonoids, EGCG without CLC regulation ability and TZD class PPARG agonists Ros and Pio have better research and development prospects.

PPARs regulate multiple biological processes, including fatty acid oxidation in peroxisomes, as an important regulatory gene. TZD class PPARG agonists have been widely used clinically as insulin sensitizers to treat type 2 diabetes. In recent years, more studies have shown that PPARG protein plays an important role in inflammation or epithelial–mesenchymal transition-related biological processes, including fibrosis, tumor development, and processes regulating TGF-β1 gene transcription and inflammation causing tissue fibrosis [8]. How PPARA expression changes PPARG expression and activity in the nephrolithiasis inflammatory fibrosis process is still unclear [20]. Here, we hypothesized that the renal calcification fibrosis process depends not only on TGF-Β1 release but also on downregulation of PPAR expression and PPARG deactivation. PPAR expression is regulated during renal calcification, inflammatory fibrosis, and EMT processes; yellow tea acts as a PPARG ligand agonist to prevent renal calcification and EMT recurrence.

We first confirmed PPAR expression changes including upregulation PPARG, TGF-β1, TNF-α, and MCP-1 expression in ethylene glycol-induced nephrolithiasis rats by RT-qPCR, immunofluorescence staining, and Western blot analyses. Upregulated collagen fibrosis was mainly located in dilated, damaged tubules at the renal cortex–medulla junction area. In addition, expression of the epithelial marker E-cadherin was increased, mesenchymal markers α-SMA and vimentin decreased, and fibronectin and collagen 1 expression levels were relatively lower in the yellow tea gavage nephrolithiasis rat model group. These results initially confirmed that EMT occurred in the rat kidney, promoting nephrolithiasis and renal fibrosis, while upregulation of PPARG activation inhibited this process.

Our results showed that yellow tea gavage can alleviate renal crystal deposition and tissue fibrosis in ethylene glycol-induced nephrolithiasis rats. In addition, yellow tea gavage also activated the rat kidney PPARG signaling pathway, inhibiting stone-induced ROS production and subsequent renal injury; it also downregulated TGF-Β1 expression, inhibiting TGF-Β1-mediated EMT. The potential mechanism of yellow tea protection involves anti-inflammatory, anti-oxidant, anti-fibrotic, and EMT inhibition mechanisms. Therefore, yellow tea may act as a potential PPARG agonist for prevention of renal oxidative damage and fibrosis caused by nephrolithiasis.

## 4. Materials and Methods

### 4.1. Animal Model of Renal Oxalate Stones and Yellow Tea Administration

All animal experiments were approved by the Animal Management Ethics and Welfare Committee of the School of Life Sciences, Fudan University. A total of 32 five-week-old male Sprague Dawley rats (weight: 130–152 g) were purchased from SLAC Animal Co., Ltd. (Shanghai, China). After a one-week acclimation period, the rats were randomly divided into four groups: control group (Ctrl), ethylene glycol-induced renal stones plus water gavage (Gly), ethylene glycol-induced renal stones plus 0.5 g/dL yellow tea infusion gavage (Gly plus YT 0.5), and ethylene glycol-induced renal stones plus 3 g/dL yellow tea infusion gavage (Gly plus YT 3). The rat gavage concentration of yellow tea was calculated as 3 g/dL according to the equivalent dose conversion table and the oral equivalent coefficient Km = 6 for rats and humans [21]. The Gly group received 1% ethylene glycol combined with 0.75% ammonium chloride drinking water for one week followed by 1% ethylene glycol drinking water and hot water gavage at 55–60 °C between 17:00 and 18:00 daily. The Gly plus YT groups received the same drinking water as the Gly group but yellow tea aqueous extract gavage at the corresponding concentration instead of water. The Ctrl group received a normal diet and water daily.

After four weeks, the different rats were housed in separate metabolic cages for 24 h, and urine samples were collected. After urine collection, the rats were anesthetized and euthanized, and blood was taken from the eye vein immediately. The plasma was prepared by centrifugation and stored at −80 °C for later use. The liver and kidney tissues were removed, washed with ice-cold phosphate-buffered saline (PBS), blotted dry, snap-frozen, and stored at −80 °C for later analysis.

### 4.2. Histological Methods to Analyze Renal Tissue Pathology

Hematoxylin and eosin (H&E) staining, Von Kossa staining, Masson’s trichrome staining, and Sirius red staining were used to stain and observe the renal tissue sections of the rats as described by a previous study [10].

### 4.3. Biochemical and Molecular Marker Measurements in Serum, Urine, and Cell Samples

Creatinine (CRE), calcium, malondialdehyde (MDA), superoxide dismutase (SOD), glutathione (GSH), and catalase (CAT) in serum, urine, and cell samples were analyzed using kits (Nanjing Jiancheng Bioengineering Institute, Nanjing, China) and a MultiSkan3 enzyme-linked immunosorbent assay instrument (Thermofisher, Shanghai, China). Oxalate (oxalic acid) in serum was tested using an oxalate colorimetric assay kit (Biovision, K663-100, Geneva, Switzerland).

### 4.4. qRT–PCR

The reliability of transcriptome sequencing was confirmed with qRT–PCR. RNA was extracted from leaves using a TRIzol kit (Accurate Biology, Changsha, China); cDNA was prepared using a reverse transcription kit (Accurate Biology, Changsha, China); and real-time PCR was performed using the SYBR Green method (Accurate Biology, Changsha, China). SiActin (SETIT_026509mg) was used as an internal standard, and relative expression levels were calculated using the 2^−ΔΔCt^ method. At least three replicates were performed in each independent experiment. Primer Premier 5 software (Primer Premier 5.0) was used to design the PCR primers.

### 4.5. Western Blotting

Rat kidney samples were lysed with RIPA lysis buffer containing protease inhibitors (Sigma-Aldrich, St. Louis, MO, USA) and phosphatase inhibitors. The protein concentration was determined by the BCA assay (Thermo Fisher, Waltham, MA, USA). The proteins in these extracts were separated by electrophoresis and transferred onto a nitrocellulose membrane (Bio-Rad, Heracles, CA, USA), which was next blocked with 1% BSA for 1 h and then incubated overnight at 4 °C with a relevant primary antibody: anti-E-cadherin antibody, anti-vimentin antibody, anti-α-smooth muscle actin (SMA) antibody, anti-peroxisome proliferator-activated receptor alpha (PPARA) antibody, anti-PPARG antibody, anti-tumor necrosis factor-alpha (TNF-α) antibody, anti-transforming growth factor-beta (TGF-β1) antibody, anti-tubulin antibody, and anti-GAPDH antibody (Abcam, Eugene, OR, USA; cat. # ab231303, ab92547, ab7817, ab227074, ab178860, ab205587, ab179695, ab7291, and ab845, respectively). After an immunoreaction with a corresponding secondary antibody (Abcam, Boston, MA, USA, cat. # ab6721), bands of proteins were detected by means of a ChemiDoc MP Imaging System (Bio-Rad, Hercules, CA, USA). GAPDH and tubulin were employed as the internal loading controls.

### 4.6. Immunofluorescence Staining

Rat kidney tissues were fixed with 4% PFA for 10 min and permeabilized with 0.1% Triton X-100 in PBS, then incubated in 5% BSA with PBS for 30 min to minimize nonspecific antibody binding. The tissues were then incubated with rat primary antibodies for 12 h at 4 °C. The following day, the tissues were incubated with goat anti-rabbit IgG labeled with Alexa 488 and goat anti-mouse IgG labeled with Alexa 594 at room temperature for 1 h. DAPI was used to stain the nuclei. The images were obtained using an LSM 510 META confocal microscope (Carl Zeiss, San Diego, CA, USA) [19].

### 4.7. Compound Structure Acquisition and Pharmacophore Prediction

The protocol for the specific component analysis of yellow tea infusion metabolites was detailed in a previous paper [19]. The compound structures were precursor conditions for predicting compound targets, and the 2D and 3D structures of all yellow tea representative compounds were obtained from PubChem (pubchem.ncbi.nlm.nih.gov/, accessed on 18 July 2023), which was used to search compounds and download 2D and 3D structure files in SDF format and graphics. Pharmmapper (lilab-ecust.cn/pharmmapper, accessed on 15 May 2022) was used to predict target compound pharmacophores.

### 4.8. Target Screening, Network Generation, and GO Analysis

String-db.org was used to draw the protein interaction network, and IID (iid.ophid.utoronto.ca/, accessed on 18 July 2023) and GeneMANIA (genemania.org, accessed on 18 July 2023) were used to verify the key protein–protein interaction (PPI) network based on string analysis. Hiplot (hiplot-academic.com/, accessed on 18 July 2023) was used to draw drug, disease target, and metabolite network maps. Metascape was used for the GO analysis of yellow tea compound targets; the yellow tea compound targets intersecting with GeneCard targets related to kidney stones, chronic kidney disease, and kidney fibrosis were obtained from a previous paper [22].

### 4.9. Screening Metabolite-Related Targets and Construction of Synergistic Network

Human Proteome Atlas (HPA, proteinatlas.org) organ-specific protein information was used to screen metabolite-related protein targets obtained from the network pharmacology and metabolomics analysis. A two-layer protein/metabolite network was constructed using the HMDB and IID databases as follows: (1) extract metabolites that interact with major target proteins; (2) extract all proteins that interact with these metabolites; and (3) add proteins that interact with the target proteins to produce a two-layer protein/metabolite network centered on the target proteins.

### 4.10. QSAR Model Validation of PPARG Agonist Activity of Flavonoids

Based on quantitative structure–activity relationship (QSAR) and random forest model training models, the PPARG agonist activity of flavonoid small-molecule compounds was predicted. PPARG was used as the protein target to retrieve the small-molecule agonist Simplified Molecular Input Line Entry System (SMILES) data from ChEMBL30. PaDEL-Descriptor was used to calculate the molecular descriptors of the small molecule agonists and the flavonoid compounds in yellow tea. R studio was used to perform data preprocessing and random forest model training using the small-molecule agonists and their molecular descriptors.

### 4.11. Cell Culture and Treatments

Human kidney proximal tubular epithelial (HK-2) cells were cultured in DMEM/F12 medium supplemented with 10% FBS and 1% penicillin/streptomycin (Gibco) and incubated in a humidified incubator at 37 °C with 5% CO_2_ (Heraeus, Hanau, Germany). Eosinophils (Eos) were purified from human blood and cultured in high-glucose DMEM medium supplemented with 10% FBS and 1% penicillin/streptomycin (Gibco). Eos cells (1 × 10^6^/mL) were added to confluent HK-2 cells and co-cultured for 24 h, as described in a previous paper [23].

Palmitate solution was prepared by dissolving palmitate completely in anhydrous ethanol and then adding it to preheated 10% FBS to bind palmitate to albumin; the final concentration of palmitate was 3 mM.

HK-2 cells were grown to confluence in 6-well plates and treated with different drugs one hour before H_2_O_2_ stimulation, as described in a previous paper [24]. The drugs were diluted in serum-free DMEM/F12 medium and added to the cells at the following concentrations: palmitate (10, 50, 100 µM), Pio (1 µM), and Ros (10 µM), as described by a previous paper [25], epigallocatechin gallate (EGCG) (100 µM; Vogda Biotechnology, Wuhan, China), isovitexin (100 µM; Vogda Biotechnology, Wuhan, China), and baicalin (100 µM; Vogda Biotechnology, Wuhan, China). H_2_O_2_ was diluted in serum-free DMEM/F12 medium to 500 µmol/L and added to the cells for one hour. 

### 4.12. Cell Viability Assay

HK-2 cells were washed twice with PBS and then cultured in complete medium containing 10% CCK-8 (Dojindo, Japan) at 37 °C for one hour to form water-soluble formazan. The absorbance at 450 nm was measured using a microplate reader (MDC). 

### 4.13. Biochemical Marker Detection

Free fatty acid (FFA) concentrations in different groups of HK-2 cells were analyzed using the corresponding kits (Nanjing Jiancheng Bioengineering Institute). A human Charcot Leyden crystal protein (CLC) ELISA kit (LMAI Bio) was used to measure CLC protein expression in co-cultured eosinophils according to the manufacturer’s instructions.

### 4.14. PPARG Transcription Factor Activity Assay

PPARG transcription factor activity was measured using a TransAM kit based on ELISA from Active Motif (Carlsbad, CA, USA), following the manufacturer’s instructions.

### 4.15. Data Analysis

Stained histological sections were analyzed using ImageJ (NIH), while the other data were analyzed using GraphPad Prism 8.0 (GraphPad, San Diego, CA, USA). All results are expressed as mean ± standard deviation (SD) and analyzed using unpaired Student’s *t*-test or one-way analysis of variance. *p*-values < 0.05 were considered statistically significant.

## 5. Conclusions

Our findings revealed the role of flavonoids as PPARG agonists for the prevention and treatment of nephrolithiasis-related oxidative damage and fibrosis. Our findings suggested that yellow tea could be a potential natural source of PPARG agonists for the prevention and treatment of nephrolithiasis. Our findings also provided new insights into the molecular mechanisms of yellow tea protection against nephrolithiasis.

## Figures and Tables

**Table 1:** Ten major functional molecules in yellow tea infusion (OB, oral bioavailability; DL, drug-likeness).

Formula	Molecule	Molecular Weight	Class	OB (%)	DL
C_22_H_18_O_11_	(-)-Epigallocatechin gallate	458.08396	Flavan-3-ol	55.09	0.77
C_21_H_18_O_11_	Baicalein 7-O-Glucuronide	446.08371	Flavone	40.12	0.75
C_21_H_20_O_10_	Isovitexin	432.10466	Flavone	31.29	0.72
C_30_H_26_O_12_	Procyanidin B1	578.14173	Flavan-3-ol	67.87	0.66
C_14_H_6_O_8_	Ellagic acid	302.00551	Organic Acid	43.06	0.43
C_15_H_10_O_7_	Quercetin	302.04179	Flavonol	46.43	0.28
C_15_H_10_O_7_	Herbacetin	302.04178	Flavonol	36.07	0.27
C_15_H_14_O_6_	Epicatechin	290.07823	Flavan-3-ol	48.96	0.24
C_15_H_10_O_6_	Kaempferol	286.04692	Flavonol	41.88	0.24
C_15_H_10_O_5_	Galangin	270.05195	Flavonol	45.55	0.21

**Table 2:** Metabolites interacting with PPARG gene (partial list).

HMDB ID	Molecular	Formula	Molecular Weight
HMDB0000039	Butyric acid	C_4_H_8_O_2_	88
HMDB0000220	Palmitic acid	C_16_H_32_O_2_	256
HMDB0000827	Stearic acid	C_18_H_36_O_2_	284
HMDB0001338	Palmityl-CoA	C_37_H_66_N_7_O_17_P_3_S	1006
HMDB0002212	Arachidic acid	C_20_H_40_O_2_	313
HMDB0002259	Heptadecanoic acid	C_17_H_34_O_2_	270
HMDB0002845	Hexanoyl-CoA	C_27_H_46_N_7_O_17_P_3_S	866

**Table 3:** Commercialized PPARG agonists (top 3) and flavonoid compounds in yellow tea and their predicted pEC50 for PPARG.

Molecule	CHEMBL ID	pEC50 (PPARG)
Rosiglitazone	CHEMBL121	7.12
Chiglitazar	CHEMBL4650349	6.68
Pioglitazone	CHEMBL595	6.60
Isovitexin	CHEMBL465360	6.29
Epigallocatechin gallate	CHEMBL297453	5.60
Baicalin	CHEMBL485818	5.57
Ellagic acid	CHEMBL6246	5.39
Procyanidin B1	CHEMBL504937	5.24
Herbacetin	CHEMBL611029	5.21
Galangin	CHEMBL309490	5.06
Kaempferol	CHEMBL150	5.06
Quercetin	CHEMBL50	5.05
Epicatechin	CHEMBL129482	4.81

## Data Availability

The data presented in this study are available upon request from the corresponding author.
